# Socioeconomic inequality in dental care utilization in Iran: a decomposition approach

**DOI:** 10.1186/s12939-019-1072-5

**Published:** 2019-10-22

**Authors:** Satar Rezaei, Mohammad Hajizadeh, Seyed Fahim Irandoost, Yahya Salimi

**Affiliations:** 1 Research Center for Environmental Determinants of Health, Health Institute, Kermanshah University of Medical Sciences, Kermanshah, Iran; 2 School of Health Administration, Faculty of Health, Dalhousie University, Halifax, Canada; 3 Social Development and Health Promotion Research Center, Health Institute, Kermanshah University of Medical Sciences, Kermanshah, Iran

**Keywords:** Socioeconomic status, inequality, Dental care utilization, Concentration index, Decomposition, Iran

## Abstract

**Purpose:**

Socioeconomic inequalities in dental care utilization in Iran are rarely documented. This study aimed to provide insight into socioeconomic inequalities in dental care utilization and its main contributing factors among Iranian households.

**Design/methodology/approach:**

A total of 37,860 households from the 2017 Household Income and Expenditure Survey (HIES) were included in the study. Data on dental care utilization, age, gender and education attainment of the head of household, socioeconomic status of households, health insurance coverage, living areas and provinces were obtained for the survey. The concentration curve and the normalized concentration index (*C*_*n*_) was used to illustrate and quantify socioeconomic inequalities in dental care utilization among Iranian households. The *C*_*n*_ was decomposed to identify the main determinants of the observed socioeconomic inequality in dental care utilization in Iran.

**Findings:**

The study indicated that the prevalence of dental care utilization among Iranian’s households was 4.67% (95% confidence interval [CI]: 4.46 to 4.88%). The results suggested a higher concentration of dental care utilization among socioeconomically advantaged households (*C*_*n*_ = 0.2522; 95% CI: 0.2258 to 0.2791) in Iran. Pro-rich inequality in dental care utilization also found in rural (*C*_*n*_ = 0.2659; 95%CI: 0.2221 to 0.3098) and urban (*C*_*n*_ = 0.0.2504; 95% CI: 0.0.2159 to 0.2841) areas. The results revealed socioeconomic status of households, age and education status of head of households and residing provinces as the main contributing factors to the concentration of dental care utilization among the wealthy households.

**Originality/value:**

This study revealed pro-rich inequalities in dental care utilization among households in Iran and its provinces. Thus, health policymakers should focus on designing effective evidence-based interventions to improve healthcare utilization among household with the older head of households, lower education status, and living in relatively poor provinces to reduce socioeconomic inequality in dental care utilization in Iran.

## Introduction

Inequalities in the utilization of dental care services are a major public health concern globally. Although individuals should receive healthcare services as needed regardless of their social and economic status [1–3], there exists clear evidence that the distribution of dental care utilization is not distributed equally across different socioeconomic groups in different countries [4–6]. A systematic and meta-analysis on 117 studies with nearly eight million participants on inequality in utilization of dental care services indicated that utilization of dental care is highly influenced by social, ethnic, economic, educational conditions [1].

The current studies [7–9] have documented the differences in the utilization of dental care across the different socioeconomic segment of the population in Iran and its different regions. Kiadaliri and colleagues [10] also highlighted inequalities in the distribution of dentists across provinces of Iran. The latter study showed that the number of dentists per 100,000 population in Tehran province was 11 times higher than that of Northern Khorasan province.

Health care in Iran is delivered by three sectors, including public (provision of all three levels of healthcare), private (mainly focus on provision secondary and tertiary level) and not-for-profit organizations. Healthcare in Iran is funded through the government’s general revenue, health insurance organizations, and out-of-pocket payments by patients [11, 12]. While the public health sector in Iran provides some dental services for rural residents, the private sector mainly provides dental care in urban areas. Of the total 20,000 dentists in Iran, roughly 90% are working in the private sector [13]. Dental care is not covered by public health insurance in Iran, and less than 20% of total dental care costs are covered by the public healthcare system [14].

Reducing socioeconomic inequality in the utilization of healthcare is a major health policy challenge in many countries [15], including in Iran. Since dental care services are not fully covered by basic health insurance coverage in Iran, these services pose a significant financial burden on patients and their households [7]. Financial barriers to receiving dental care in Iran may have also resulted in unmet dental healthcare services among socioeconomically disadvantaged households. To date, the current literature highlighted the issue of socioeconomic inequalities in access and utilization of dental care in developed countries [2–4, 16]. Although some studies in Iran [7–9] indicated the role of socioeconomic status on the utilization of dental care in some provinces in Iran, no study examines socioeconomic inequality in the utilization of dental care in Iran and across its provinces. Therefore, this study, for the first time, aimed to measure socioeconomic inequality in dental care utilization and its main determinants in Iran using the concentration index approach. The results of this study provide useful information for policymakers to design effective policies to reduce socioeconomic inequalities in the utilization of dental care in Iran.

## Methods and materials

### Study setting

Iran is an upper-middle-income country located in the Eastern Mediterranean Region (EMR), with an area of 1,648,000 km^2^. According to the 2016 census data, the population of Iran was approximately 80 million people living across 31 provinces. Almost 76% of the Iranian population live in urban areas.

### Data and variables

Data were derived from the 2017 Household Income and Expenditure Survey (HIES), which collected annually by the Iranian Statistical Center (ISC) (https://www.amar.org.ir/english/Statistics-by-Topic/Household-Expenditure-and-Income#2220530-releases). The survey collects information through adirect interview with the household head. The questionnaire used to collect data in the HIES was designed under supervisions and recommendations of the United Nations. The target population of the survey includes all households occupying a private dwelling and a collective dwelling in urban and rural areas in Iran. The sampling procedure used in the survey is a three-stage cluster sampling method with strata. At the first stage, the census areas are classified and selected. The urban and rural blocks are selected in the second stage. In the final stage, households in the sample were selected (https://www.amar.org.ir/english/Statistics-by-Topic/Household-Expenditure-and-Income#2220530-releases). Data on sociodemographic of household (e.g., the age of head of household, the gender of head of household, education status of head of household and household size), household healthcare utilization, household’s assets, income and expenditure were collected using a face-to-face interview with the head of households. In 2017, data were collected from 37,860 households in rural and urban areas of Iran.

The outcome variable in the study is a binary variable indicating whether or not the household used any dental care services (e.g., visit, extraction and restoration, prosthodontics, orthodontics in governmental and non-governmental centers) in the last month. Based on the previous studies [1, 2, 4, 8, 9] and availability of information in the HEES, the following factors used as determinants of dental care utilization: age of head of household, gender of head of household, education status of head of household, a constructed wealth index of households (as a proxy for socioeconomic status), health insurance coverage and living areas (urban/rural). Provincial fixed effects were also included to control for any differences across provinces.

The principal component analysis (PCA) technique [17, 18] was used to construct a wealth index for households. As per the current literature [17, 19, 20], housing characteristics (rooms per capita, type of house ownership, house size per square meter) and durable assets of households (e.g., car, TV color, internet, computer/laptop, cell phone, freezer, dishwasher, microwave, vacuum cleaner, motorcycle and bicycle) were used in the PCA. Based on the wealth scores obtained from the PCA, households were classified into five socioeconomic status (SES) groups from the poorest (first quintile) to the richest (fifth quintile).

### Statistical analysis

#### Measuring socioeconomic inequality in dental care utilization

The concentration curve and the concentration index (*C*) [21–23] were used to assess socioeconomic-related inequalities in dental care utilization among households in Iran and its provinces. The concentration curve is a graph that depicts the cumulative proportion of a health outcome in y-axis against the cumulative proportion of the population ranked by a socioeconomic status (SES) indicator in the x-axis. If the concentration curve lies above the 45-degree line (perfect equality line), it suggests that the health outcome is concentrated among the low SES individuals and vice versa. The value of C varies between − 1 and + 1, with the value zero indicating “perfect equality”. A positive (negative) sign of the *C* shows the outcome variable (e.g., dental care utilization) is more concentrated among socioeconomically advantaged (disadvantaged) households. Since the outcome variable in this study is binary, the estimated *C* is not between − 1 and + 1. Thus, as suggested by Wagstaff [24], we normalized the *C* by dividing the estimated it by $\frac{1}{1-\upmu}$, where μ is the mean of the outcome variable.

#### Decomposing socioeconomic inequality in dental care utilization

The *C* can be decomposed to identify the main determinants of observed socioeconomic inequality in dental care utilization. If there is a regression model that links the dental care utilization, *y*, to a set of explanatory variables *x*_*k*_ as:

1 $$ \mathrm{y}=\upalpha +\sum \limits_k{\beta}_k\ {x}_k+\varepsilon . $$

The *C* for dental care utilization, *y*, can be decomposed using the following equation [25]:

2 $$ C=\sum \limits_k\left(\frac{\beta_k{\overline{x}}_k}{\mu}\right){C}_k+\frac{G{C}_{\varepsilon }}{\mu }, $$

where ${\overline{x}}_k$ is the mean of determinants *x*_*k*_, *C*_*k*_ is the *C* for explanatory factors *x*_*k*_, $\left(\frac{\beta_k{\overline{x}}_k}{\mu}\right){C}_k$ is the elasticity of dental care utilization with respect to the explanatory variable *x*_*k*_. Elasticity shows the amount of change in dependent variable associated with a one-unit change in the explanatory variable. A negative (positive) elasticity for an explanatory variable in our study indicates that an increase in an explanatory variable decreases (increases) the probability of dental care utilization. The $\sum \limits_k\left(\frac{\beta_k{\overline{x}}_k}{\mu}\right){C}_k$ presents the contribution of explanatory factor, *x*_*k*_, to the overall *C* for dental care utilization. The last term, $\frac{G{C}_{\varepsilon }}{\mu }$, is the residuals component and shows the portion of the *C* which cannot be explained by the variables included in the model. The normalized concentration index for dental care utilization, *C*_*n*_, can be decomposed as:

3 $$ {C}_n=\frac{C}{1-\mu }=\frac{\sum \limits_k\left(\frac{\beta_k{\overline{x}}_k}{\mu}\right){C}_k}{1-\mu }+\frac{\frac{A{C}_{\varepsilon }}{\mu }}{1-\mu } $$

Based on Eq. 3, the absolute contribution for an explanatory factor was calculated by multiplying the elasticity of dental care utilization with respect to the explanatory variable by the *C* for the explanatory factor and dividing it by 1 − *μ*. The absolute contribution shows how much of the association between wealth status and dental care utilization is explained by variation in a given explanatory factor across socioeconomic groups. A negative absolute contribution of an explanatory factor to the *C*_*n*_ suggests that the socioeconomic distribution of that factor and the association of the relevant factor with dental care utilization contributes to lower utilization of dental care among poorer households and vice versa. The relative contribution of each explanatory factor was computed by dividing the absolute contribution for each explanatory factor by the *C*_*n*_ and then multiplying by 100.

As dental care utilization is a binary variable, the marginal effect of explanatory factors obtained from non-linear logit regression was used as *β*_*k*_ in the decomposition analysis. All the analyses were performed using Stata Version 14. *P*-values less than 0.05 considered statistically significant.

## Results

### Descriptive statistics

The descriptive characteristics of the study population are reported in Table 1. Of the 37,860 households included in the study, 86.1% of the households headed by men. The average age of the head of households was 51.7 years (standard deviation [SD] = 15.5). Approximately, 85 % of the head of households were married, and 72.8% of head of households were literate.

**Table 1:** Descriptive characteristics of households included in the analysis, 2017

Variables	*n* (%)	Percentage of households with dental care utilization in the last month (95% confidence interval)
Demographic variables		
*Sex of household head*		
Male	32,595 (86.1)	5.1 (4.87 to 5.34)
Female	5265 (13.9)	1.97 (1.63 to 2.38)
*Age of household head*		
15–45	15,152 (40)	5.47 (5.12 to 5.84)
46–65	15,163 (40.1)	5.1 (4.73 to 2.59)
66 and above	7545 (19.9)	2.23 (1.92 to 2.59)
Socioeconomic variables		
*Marital status of household head*		
Single	5351 (14.1)	5.14 (4.91 to 5.39)
Married	32,049 (84.7)	2.03 (1.69 to 2.45)
Divorced/widow	460 (1.2)	2.17 (1.17 to 4.00)
*Education status of household head*		
Illiterate	10,287 (27.2)	5.64 (5.38 to 5.92)
Literate	27,573 (72.8)	2.06 (1.80 to 2.35)
*Wealth index of households*		
Poorest	7529 (19.9)	1.73 (1.46 to 2.06)
Poor	7569 (20)	3.32 (2.94 to 3.75)
Middle	7585 (20)	5.08 (4.61 to 5.60)
Rich	7586 (20)	5.47 (4.98 to 6.06)
Richest	7591 (20.1)	7.69 (7.11 to 8.31)
*Insurance coverage*		
Yes	33,674 (88.9)	4.78 (4.56 to 5.02)
No	4186 (11.1)	3.72 (3.19 to 4.34)
Ecological variables		
*Geographical area*		
Urban	18,655 (49.3)	5.96 (5.63 to 6.30)
Rural	19,205 (50.7)	3.41 (3.16 to 3.68)
*Province*		
Tehran	1947 (5.1)	5.03 (4.14 to 6.09)
Markazi	1447 (3.8)	3.66 (2.80 to 4.76)
Gilan	1211 (3.2)	4.04 (3.06 to 5.31)
Mazandaran	1104 (2.9)	4.80 (3.68 to 6.23)
East Azerbaijan	1278 (3.4)	9.85 (8.33 to 11.62)
West Azerbaijan	1147 (3.0)	2.70 (1.90 to 3.81)
Kermanshah	1188 (3.1)	6.56 (5.28 to 8.12)
Khuzestan	1356 (3.6)	2.80 (2.04 to 3.82)
Fars	1485 (3.9)	5.85 (4.77 to 7.17)
Kerman	1155 (3.1)	1.56 (0.98 to 2.5)
Razavi Khorasan	1567 (4.1)	5.68 (4.63 to 6.94)
Esfahan	1345 (3.6)	9.56 (8.13 to 11.28)
Sistan and Baluchestan	1386 (3.7)	0.94 (0.54 to 1.60)
Kurdistan	881 (2.3)	4.43 (3.25 to 6.00)
Hamadan	1260 (3.3)	4.84 (3.78 to 6.17)
Chahar Mahall and Bakhtiari	924 (2.4)	7.14 (5.65 to 8.99)
Lorestan	1068 (2.8)	4.21 (3.15 to 5.59)
Ilam	998 (2.6)	4.00 (2.95 to 5.42)
Kohgiluyeh Buyer Ahmad	1130 (3.0)	6.81 (5.48 to 8.44)
Bushehr	1124 (3.0)	2.40 (1.65 to 3.48)
Zanjan	1116 (2.9)	3.67 (2.71 to 4.95)
Semnan	959 (2.5)	4.69 (3.51 to 6.23)
Yazd	1215 (3.2)	4.27 (3.27 to 5.57)
Hormozgan	1419 (3.7)	7.54 (6.27 to 9.03)
Ardebil	1024 (2.7)	4.00 (2.59 to 5.39)
Qom	1026 (2.7)	6.82 (5.43 to 8.54)
Qazvin	1006 (2.7)	2.78 (1.92 to 4.00)
Golestan	1344 (3.5)	2.60 (1.87 to 3.60)
North Khorasan	1394 (3.7)	4.66 (3.67 to 5.90)
South Khorasan	1321 (3.5)	2.12 (1.46 to 3.05)
Alborz	1035 (2.7)	3.76 (2.76 to 5.11)

**Fig. 1:**
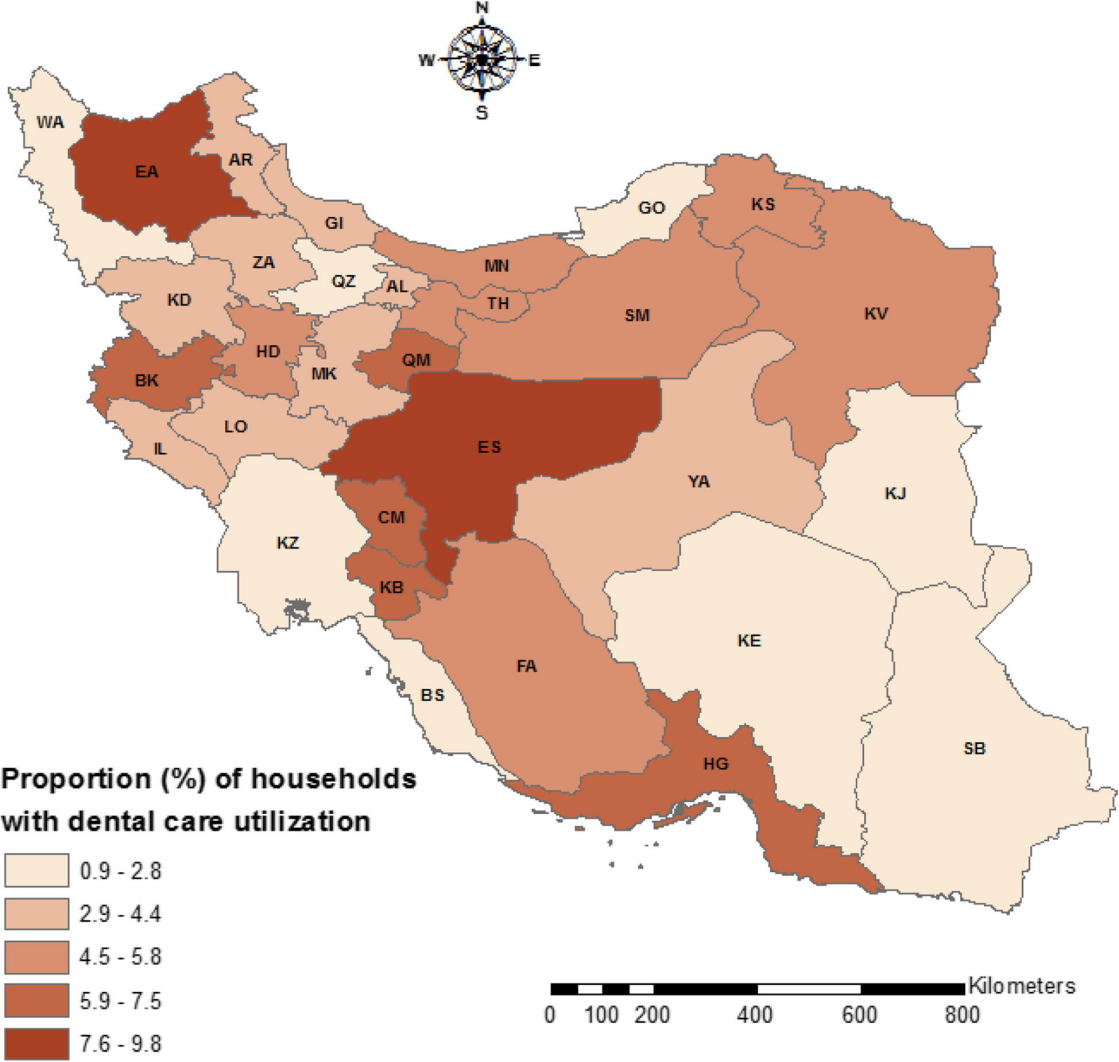
Proportion of households with dental care utilization over the past month across provinces in Iran, 2017

On average, 4.67% (95% confidence interval [CI]: 4.46 to 4.88%) of households used dental care services in the last month. There is a large variation among the provinces in dental care utilization in Iran (see Fig. 1). While less than 1% of the households in Sistan and Baluchestan province used dental care in the past month, this figure was more than 9% in East Azarbaijan and Esfahan provinces. The proportion of households with any dental care utilization varied across different socioeconomic groups of households. The proportion of any dental care utilization among households was 1.73 and 7.69% among the poorest and the wealthiest SES groups, respectively (see Table 1).

### Socioeconomic inequality in dental care utilization

**Fig. 2:**
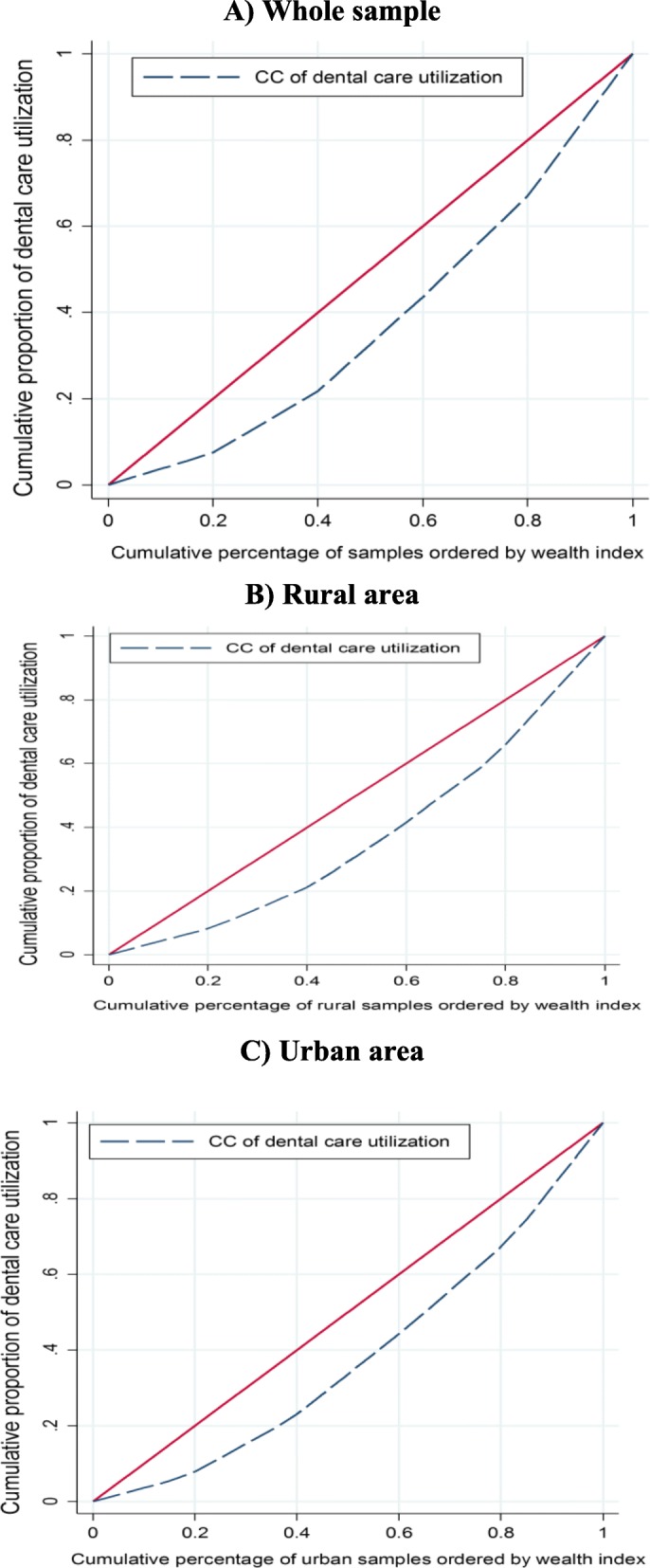
The Concentration curve for dental care utilization in Iran for whole sample, rural and urban areas

Figure 2 reports the concentration curve for dental care utilization among households in last month in Iran as well as in urban and rural areas. As illustrated in the figure, the concentration curves lie below the 45-degree line indicating the concentration of dental care utilization among the wealthy households.

Based on the results, dental care utilization is more concentrated among socioeconomically advantaged households (*C*_*n*_ = 0.2522; 95%CI = 0.2258 to 0.2791). Pro-rich inequality in dental care utilization is also found in rural (Cn = 0.2659; 95% CI = 0.2221 to 0.3098) and urban (Cn = 0.0.2504; 95% CI = 0.0.2159 to 0.2841) areas (see Table 2).

**Table 2:** The normalized concentration indices for dental care utilization for whole sample, and rural and urban areas in Iran

Sample	Samples	The *C*_*n*_	95% Confidence interval
Urban	18,655	0.2504	0.2159 to 0.2841
Rural	19,205	0.2659	0.2221 to 0.3098
Total	37,860	0.2522	0.2258 to 0.2791

**Fig. 3:**
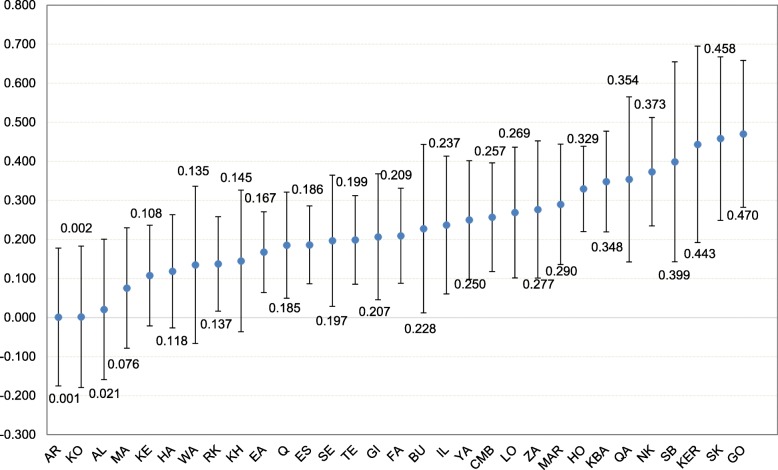
The normalized concentration index for dental care utilization across Iranian provinces, 2017

As illustrated in Fig. 3, there existed a significant pro-rich inequality in dental care utilization among households in the majority of provinces in Iran. The highest and lowest socioeconomic inequality in dental care utilization were found in Ardabil (*C*_*n*_ = 0.0012) and Golestan (*C*_*n*_ =0 .4702) provinces, respectively.

### Determinants of socioeconomic inequality in dental care utilization

Table 3 reported the results of the decomposition analysis of socioeconomic inequality in dental care utilization among households in Iran. Based on the results of marginal effects of determinants, the probability of dental care utilization was higher in male-headed households, households with the younger head of household, households with a married head of household. The probability of dental care utilization was also higher among urban households as compared to their rural household counterparts. A positive association existed between having health insurance coverage and the use of dental care. The probability of dental care utilization among the highest SES household group was 3.7% higher than the lowest SES household group.

**Table 3:** Decomposition of socioeconomic inequality in dental care utilization among Iranian households, 2017

	Marginal effect	Elasticity	*C*_*x*_	Contribution to the *C*_*n*_		
				Contribution	%	Summed percentage
Demographic variables						
*Sex of household head*						
Male	0.0144^**^	0.2655	0.0511	0.0142	5.65	5.65
Female (ref.)						
*Age of household head*						
15–45(ref.)						
46–65	−0.0022	− 0.0188	0.0813	− 0.0016	− 0.64	
66 and above	−0.0175^***^	− 0.0748	− 0.2034	0.0160	6.32	5.69
Socioeconomic variables						
*Marital status of household head*						
Single (ref.)						
Married	0.0017	0.0312	0.0589	0.0019	0.76	
Divorced/widow	−0.0204^*^	− 0.0053	− 0.3004	0.0017	0.66	1.42
*Education status of household head*						
Illiterate (ref.)						
Literate	0.0114^***^	0.1781	0.1068	0.0200	7.91	7.91
*Wealth index of households*						
Poorest (ref.)						
Poor	0.0135^***^	0.0577	−0.4024	− 0.0243	−9.65	
Middle	0.0245^***^	0.1050	−0.0021	− 0.0002	− 0.09	
Rich	0.0254^***^	0.1091	0.3986	0.0456	18.08	
Richest	0.0377^***^	0.1619	0.7995	0.1358	53.82	62.16
*Insurance coverage*						
Yes	0.0065^**^	0.1243	0.0134	0.0017	0.69	0.69
No (ref.)						
Ecological variables						
*Geographical area*						
Urban	0.0187^***^	0.1977	−0.0926	− 0.0192	−7.62	−7.62
Rural (ref.)						
*Province*						
Tehran (ref.)						
Markazi	−0.0289^***^	− 0.0236	0.0122	−0.0003	− 0.12	
Gilan	−0.0240^***^	− 0.0165	− 0.1053	0.0018	0.72	
Mazandaran	−0.0256^***^	−0.0160	0.1759	−0.0029	−1.17	
East Azerbaijan	0.0036	0.0026	0.0512	0.0001	0.06	
West Azerbaijan	−0.0433^***^	−0.0281	0.0578	−0.0017	− 0.68	
Kermanshah	−0.0036	−0.0024	− 0.2321	0.0006	0.24	
Khuzestan	−0.0372^***^	−0.0286	− 0.1917	0.0057	2.28	
Fars	−0.0164^***^	−0.0138	0.0813	−0.0012	− 0.47	
Kerman	−0.0524^***^	−0.0342	− 0.3863	0.0139	5.50	
Razavi Khorasan	−0.0154^***^	−0.0136	− 0.0319	0.0005	0.18	
Esfahan	−0.0009	−0.0007	0.2906	−0.0002	− 0.08	
Sistan and Baluchestan	−0.0635^***^	−0.0498	− 0.6079	0.0318	12.59	
Kurdistan	−0.0218^***^	−0.0108	− 0.0637	0.0007	0.29	
Hamadan	−0.0243^***^	−0.0173	0.0930	−0.0017	− 0.67	
Chahar Mahall and Bakhtiari	−0.0110^**^	−0.0058	0.1911	−0.0012	− 0.46	
Lorestan	−0.0221^***^	−0.0133	− 0.1361	0.0019	0.75	
Ilam	−0.0211^***^	−0.0119	− 0.2096	0.0026	1.04	
Kohgiluyeh Buyer Ahmad	−0.0083	−0.0053	0.0820	−0.0005	− 0.18	
Bushehr	−0.0462^***^	−0.0294	0.0169	−0.0005	− 0.21	
Zanjan	−0.0305^***^	−0.0192	− 0.0391	0.0008	0.31	
Semnan	−0.0222^***^	−0.0121	0.1030	−0.0013	− 0.52	
Yazd	−0.0325^***^	−0.0223	0.3508	−0.0082	−3.26	
Hormozgan	−0.0273^***^	−0.0219	0.2039	−0.0047	−1.86	
Ardebil	−0.0276^***^	−0.0160	− 0.0177	0.0003	0.12	
Qom	−0.0117^**^	−0.0068	0.1966	−0.0014	− 0.56	
Qazvin	−0.0400^***^	−0.0228	0.1218	−0.0029	−1.15	
Golestan	−0.0418^***^	−0.0318	0.0057	−0.0002	− 0.07	
North Khorasan	−0.0203^***^	−0.0160	− 0.0823	0.0014	0.55	
South Khorasan	−0.0474^***^	−0.0354	− 0.0166	0.0006	0.24	
Alborz	−0.0317^***^	−0.0185	0.2648	−0.0052	−2.04	11.37
Residual				0.03		12.72
The *C*_*n*_				0.25		100.0

Note: ***Significant 1%; **Significant 5%; ^*^ Significant at 10%

The results of the concentration index for the explanatory variables suggested that male-headed households, households with the younger head of household, households with the married head of households, households with the literate head of household and household with health insurance coverage were relatively wealthier in Iran. In contrary, households residing in urban areas, those of 66 years and above and households with divorced/widowed head of household were relatively poor in Iran.

The absolute and percentage contribution of explanatory variables to the socioeconomic inequality in dental care utilization in Iran are reported in the fifth column of Table 3. The decomposition results indicated that the wealth index of households, independently from other determinants, associated positively with dental care utilization. The wealth index of households explained 62.16% of the overall *C*_*n*_ for dental care utilization in Iran, suggesting that if the wealth index were equally distributed among the households, socioeconomic inequality in dental care utilization would have been decreased by 62.16%.

Other factors that contributed to the concentration of dental care utilization among socioeconomically advantaged Iranian households were the age of head of household, the gender of head of household, health insurance coverage, educational status of head of household, marital status of head of household and province residing of households.

Turning to the geographic factors, it is apparent that provinces like Sistan and Baluchestan and Kerman contribute positively to the inequality in dental care in Iran. The positive absolute contribution of these provinces to the inequality is due to the fact that, compared to residents of the capital province (Tehran), the probability of dental care use is lower among the residents of Sistan and Baluchestan and Kerman provinces (thus, it has a negative elasticity) and household living in these two provinces are relatively poorer in Iran (see the negative sign of the *C*_*k*_ for these two provinces in Table 3). The product of these two effects leads to the positive contribution of Sistan and Baluchestan and Kerman to the overall *C*_*n*_.

As shown in Tables 3, 87.28% of socioeconomic-related inequality in dental care utilization was explained by the explanatory variables included in the model. The remaining 12.72% of the inequality in dental care utilization are associated with other variables that are not included in the analysis.

## Discussion

Equal access to healthcare services such as dental care is defined as one of the main goals in all healthcare systems [1, 2]. Nevertheless, utilization of healthcare has been influenced by a variety of sociodemographic and economic factors. Previous studies reported socioeconomic inequalities in dental care utilization in high-income countries [1, 4]. A limited number of studies [7–9] also suggested the effect of socioeconomic status in the utilization of dental care in some provinces in Iran. This study, for the first time, measures the extent of socioeconomic inequalities in Iran and across 31 provinces. Further, the observed socioeconomic inequality in dental care utilization was decomposed to identify the main determinates such inequality among Iranian households.

The results revealed that there are pro-rich inequalities in dental care utilization among households in Iran and its provinces. Socioeconomic-related inequalities in dental care utilization favoring the rich are also documented in the previous national and international studies [2, 4, 7]. A study by Listl also showed pro-rich inequality in dental care utilization among Europeans adults aged 50 years and older [4]. The pro-rich inequality in dental care utilization can be explained by the fact that higher SES individuals afford to use expensive dental care due to their higher ability-to-pay and better health insurance coverage [26]. Dental care services are not fully covered by health insurance in Iran and households should pay high out-of-pocket to receive dental care services. Thus, the pro-rich inequality in dental care utilization can be partially explained by the affordability of dental treatment costs among wealthy households. A study conducted by Lutfyyia and collogues [27] among adults in the USA indicated that being uninsured and living in rural area, and a lower annual income were significantly associated with not having a dentist visit in the last 12 months.

Our decomposition analysis indicated that SES of households, itself, is the main factor contributing to the concentration of dental care utilization among the rich. Duncan and Bonner reported that low income and poverty as the main factors contributed to the observed inequality in dental care utilization in Canada [28]. Besides SES, the age of head of household, the gender of head of household, health insurance coverage, educational status of head of household, marital status of head of household and province residing of households were other factors that contributed to the concentration of dental care utilization among the wealthy households in Iran.

Our findings also showed the higher probability of dental care use among households with a younger head of household, male-headed household, married head of household, higher educational status of head of household. A systematic review and meta-analysis of the current studies demonstrated that higher educational attainment and income have a statistically positive impact on dental care utilization [1]. A previous study [7] conducted in Iran also showed a positive association between higher education status and dental care utilization, while having divorced or widowed head of household negatively associated with dental care utilization. Rezaei and colleagues [9] also founded a positive association between higher income and education attainment level and dental care utilization in Kermanshah city, western Iran. Our study also revealed that having health insurance coverage and living in urban areas increased the probability of dental care utilization among Iranian households. The latter results are similar to the findings of previous studies [1, 8, 9, 16, 28–30]. For example, Ayo-Yusuf and colleagues [31] investigated inequality in the preventive dental visits in South Africa in 2003 and 2004 and found that having health insurance coverage and living in urban areas increased the probability of the utilization of these services. Khan and colleagues [32] found that those who are living in rural areas had significantly lower odds of visiting a dentist for a checkup in the last 12 months.

Our study provides useful and important information about socioeconomic-related inequality in dental care utilization among Iranian households. However, there were some limitations that should be considered when interpreting the findings. First, the study is a cross-sectional in design; thus, we cannot establish any causal effect between dental care utilization and its determinants. Second, data on dental care utilization was collected are self-reported and may introduce some systematic error such as recall bias. Third, other factors such as the number of dentists in each province and oral health status might be affecting dental care utilization. These factors are not included in the analysis because of unavailability data in the HIES. Fourth, as the HIES collects information at the household level, we could not assess socioeconomic inequalities in dental care utilization for men and women, separately. Further studies measuring socioeconomic inequalities in dental care by sex can provide further insights into equity in dental care utilization in Iran.

## Conclusion

This study revealed that dental care utilization is more concentrated among socioeconomically advantaged households in Iran and its provinces. The findings suggested SES of households, residing provinces, age and education status of head of households as the main contributing factors to the concentration of dental care utilization among the wealthy households. Effective evidence-based interventions should be implemented to improve dental care utilization among households with the older head of households, lower education status, and living in relatively poor provinces to reduce socioeconomic inequality in dental care utilization in Iran.

## Data Availability

The data used in the study was extracted from the 2017 Household Income and Expenditure Survey (HIES) collected by the Iranian Statistical Center (ISC). The HIES are publicly available at https://www.amar.org.ir/english/Statistics-by-Topic/Household-Expenditure-and-Income#2220530-releases.
